# Electroacupuncture for women with stress urinary incontinence: Protocol for a systematic review and meta-analysis

**DOI:** 10.1097/MD.0000000000009110

**Published:** 2017-12-08

**Authors:** Weixin Huang, Xiaohui Li, Yuanping Wang, Xia Yan, Siping Wu

**Affiliations:** a The Second Affiliated Hospital of Guangzhou University of Chinese Medicine; b The Second Clinical College of Guangzhou University of Chinese Medicine, Guangdong Provincial Hospital of Chinese Medicine, Guangzhou, China.

**Keywords:** electroacupuncture, protocol, stress urinary incontinence, systematic review

## Abstract

**Background:**

Stress urinary incontinence (SUI) is a widespread complaint in the adult women. Electroacupuncture has been widely applied in the treatment of SUI. But its efficacy has not been evaluated scientifically and systematically. Therefore, we provide a protocol of systematic evaluation to assess the effectiveness and safety of electroacupuncture treatment on women with SUI.

**Methods:**

The retrieved databases include 3 English literature databases, namely PubMed, Embase, and Cochrane Library, and 3 Chinese literature databases, namely Chinese Biomedical Literature Database (CBM), China National Knowledge Infrastructure (CNKI), and Wanfang Database. The randomized controlled trials (RCTs) of the electroacupuncture treatment on women with SUI will be searched in the above-mentioned databases from the time when the respective databases were established to December 2017. The change from baseline in the amount of urine leakage measured by the 1-hour pad test will be accepted as the primary outcomes. We will use RevMan V.5.3 software as well to compute the data synthesis carefully when a meta-analysis is allowed.

**Results:**

This study will provide a high-quality synthesis to assess the effectiveness and safety of electroacupuncture treatment on women with SUI.

**Conclusion:**

The conclusion of our systematic review will provide evidence to judge whether electroacupuncture is an effective intervention for women with SUI.

**PROSPERO registration number:**

PROSPERO CRD42017070947

## Abstract 2

Supplemental Digital Content is available in the text

## 1 Introduction

Stress urinary incontinence (SUI), a common disease among women, refers to the presence of involuntary leakage of urine from the external urethral orifice when the abdominal pressure increases with a sneeze, cough, or exercise.^[1]^ Epidemiological studies showed that there are large discrepancies in terms of the prevalence rate of the disease in different cases, which may be associated with the definition of incontinence, measurement methods, the characteristics of research subjects and investigation methods, and other factors. Twenty-three percent to 45% of the female population suffer from different degrees of urinary incontinence, 7% have obvious symptoms of urinary incontinence, of whom about 49% have SUI.^[2–4]^ SUI can cause severe psychological burden and decline of life quality, bringing troubles to the patients’ family life and social activities.^[5–7]^

The American Urological Association (AUA) currently recommends a main conservative treatment, namely pelvic floor muscle training (PFMT), for mild and moderate SUI patients.^[3]^ It is reported that the short-term efficiency of PFMT can be up to 50% to 75%.^[8]^ However, there are still shortcomings, such as the poor compliance and difficulties of mastering training techniques. Due to lack of skilled trainers, the penetration rate of the PFMT is not high in China.^[9]^ Surgery is effective for the patients with severe SUI that has seriously affected their quality of life, but the treatment will cause potential complications, including pain, infection and dysuria, etc., so some may think the therapy unacceptable.^[10]^ Therefore, we need to seek other effective and safe treatments.

Originated in ancient China, acupuncture, especially the electroacupuncture, is recommended by the National Institutes of Health (NIH) as a supplementary or alternative treatment for various diseases, including SUI.^[11]^ Electroacupuncture is a form of acupuncture with electrical impulses passing through the needles to stimulate.^[12,13]^ There are researches suggesting that sacral acupuncture could help improve the acetic acid induced bladder irritation by inhibiting the capsaicin-sensitive C-fiber activation.^[14]^ In general, it is not clear how the acupuncture works (e.g., does it work through nerves, muscle, and blood?) based on current research. However, electroacupuncture has been widely used clinically by practitioners of traditional Chinese Medicine to treat SUI in China and the efficacy is satisfactory.^[7,15]^

In a randomized controlled trial (RCT),^[16]^ which was recently published in the Journal of the American Medical Association (JAMA), 504 female SUI patients were randomly divided into 2 groups: the electroacupuncture group and false electroacupuncture group (252 patients in each group), who were treated by electrically acupuncturing the acupoint Zhongliao (BL33) on both sides and Huiyang (BL35), and false electroacupuncture, respectively, no less than 18 times during 6 weeks of treatment. The results showed that the decrease of urination leakage and improvement of the quality of life in the electroacupuncture group were better than that of the false electroacupuncture group, and the difference between the groups was clinically significant.

Although many studies have demonstrated that acupuncture has a significant curative effect on SUI, its efficacy remains controversial. For instance, a meta-analysis published in 2013 suggested that acupuncture had an uncertain curative effect on SUI.^[11]^ However, the study adopted the standard interventions, including manual acupuncture (MA), scalp acupuncture, electroacupuncture, body acupuncture, and other acupuncture methods. According to 1 publication,^[17]^ electroacupuncture and MA treatment are not interchangeable. Therefore, they must be determined and studied separately for accurate research. It goes against the homogeneity of the studies of acupuncture efficacy to mix MA and electroacupuncture in the study of systematic evaluation. As far as we know, there is a huge number of clinical reports on the electroacupuncture treatment of women with SUI but lack of systematic evaluation/meta-analysis of its efficacy.

This study adopts the method of evidence-based medicine to analyze and evaluate clinical RCTs published globally on women with SUI, in order to provide evidence for further enhancing the clinical curative effect on women with SUI. The study will answer 2 questions: Is electroacupuncture an effective and safe treatment for women with SUI? If yes, is it the therapeutic effect or placebo effect?

## 2 Methods

### 2.1 Inclusion criteria for study selection

#### 2.1.1 Types of studies

All the RCTs of electroacupuncture for the management of women with SUI patients will be included without publication status restriction or writing language.

#### 2.1.2 Types of patients

All cases included in the trial will be women with SUI and the diagnoses will be based on the diagnostic criteria of the International Continence Society without any age and race limit.

#### 2.1.3 Types of interventions

The treatment group will be treated with electroacupuncture (there is no limit on the needle materials, choice of acupoints, treatment methods, retaining time of needles, and course of treatment), while the control group will adopt the internationally recognized therapy (such as PFMT and taking western medicine, etc.) or false electroacupuncture therapy.

#### 2.1.4 Types of outcome measures

##### 2.1.4.1 Primary outcomes

The primary outcome is the change from baseline in the amount of urine leakage measured by the 1-hour pad test.

##### 2.1.4.2 Secondary outcomes

- The International Consultation on Incontinence Questionnaire-Urinary Incontinence (ICIQ-UI) Short Form score;
- Self-report assessment of therapeutic effect;
- Severity of urinary incontinence;
- A 72-hour incontinence episode frequency;
- Frequency and nature of adverse events.

### 2.2 Search methods for the identification of studies

The retrieved databases include 3 English literature databases, namely PubMed, Embase, and Cochrane Library, and 3 Chinese literature databases, namely Chinese Biomedical Literature Database (CBM), China National Knowledge Infrastructure (CNKI), and Wanfang Database. The RCTs of the electroacupuncture treatment on women with SUI will be searched in the above-mentioned databases from the time when the respective databases were established to December 2017. The keywords and uncontrolled terms will be combined to retrieve data. The retrieval strategy will be determined after several pre-retrievals. The search terms include electroacupuncture, SUI and RCTs. Meanwhile, the references included in the study and the original literature included in the related system evaluation will be added to supplement the relevant literature to guarantee the recall rate. The detailed strategies for searching the PubMed database will be presented in Appendix 1 and modified by using other databases.

#### 2.2.1 Searching other resources

We will also manually retrieve the relevant conference papers, and contact the experts in the field and corresponding author to obtain important information that is not available through the previously mentioned searching.

### 2.3 Data collection and analysis

#### 2.3.1 Selection of studies

**Figure 1:**
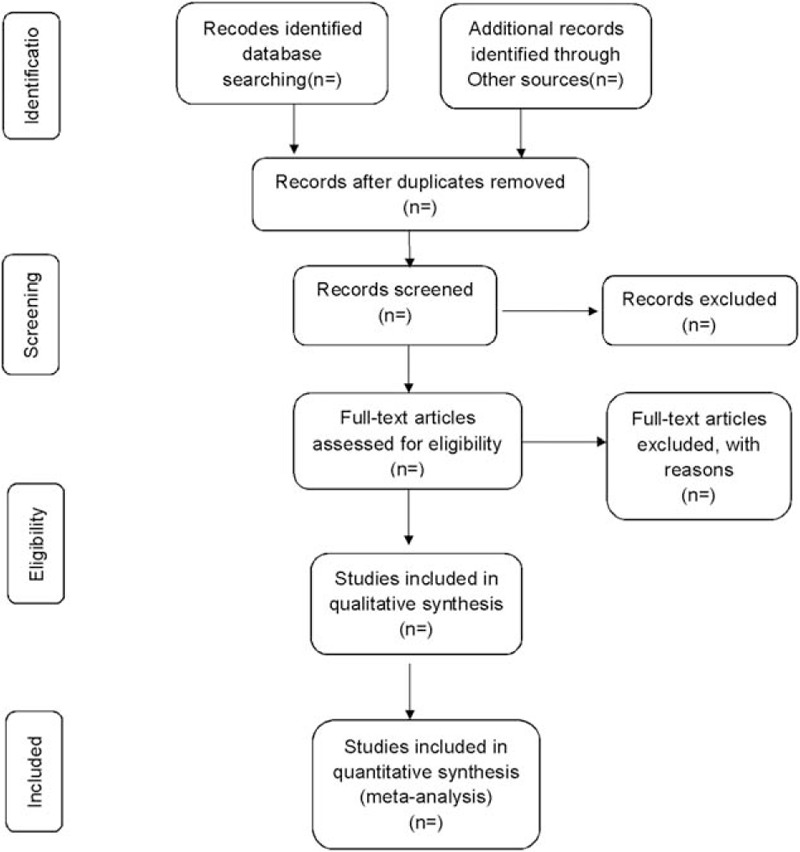
Flow diagram of study selection process.

The corresponding researchers will import the literature retrieved to the literature management system of EndnoteX7 and eliminate the duplicate data. Then, the articles that are obviously below the standard will be removed by reading the title and abstract. Later, the researchers will read the full text, discuss in the group, and contact the author to learn about the details of the study to determine the final inclusion of the literature (Fig. 1). The final list of the included articles will be transferred into Microsoft Excel format. Two researchers will independently conduct the literature search and literature screening. Finally, another study member will resolve the inconsistencies and check the final literature that will be included.

#### 2.3.2 Data extraction and management

The data will be extracted with the software EpiData 3.1 (EpiData Association, Odense, Denmark) for double entry, and the data consistency and final database will be checked by another researcher. Extracted data will be as follows: disease diagnosis, diagnosis/screening tools of SUI, coexistent disease, course of disease, phase of disease, severity of disease, sample size, age, intervention, the details of the control group, follow-up, outcome indicators, research results, adverse events, and other detail information. Where there are missing data, errors, and uncertainties, the problems will be resolved through group discussions, contacting authors, and/or third-party arbitration.

#### 2.3.3 Assessment of risk of bias in included studies

According to the Cochrane Collaboration's tool for assessing risk of bias in randomized trials provided by *Cochrane Handbook for Systematic Reviews of Interventions*, the risks will be evaluated from 7 dimensions: random sequence generation, allocation concealment, blinding method for patients, researchers and outcomes assessors, incomplete result data, and selective reports. The results of the evaluation will be divided into 3 levels, namely low-risk, unclear, and high-risk.^[18]^ The evaluation will be conducted independently by 2 trained researchers in the team. The inconsistencies will be resolved through group discussion, contacting authors, and third-party arbitration.

#### 2.3.4 Measures of treatment effect

The relative risk (RR) will be used to evaluate the enumeration data, and the mean difference (MD) will be used to evaluate the measurement data. The effect sizes will be presented with a 95% confidence interval (95% CI) for analysis.

#### 2.3.5 Dealing with missing data

As for the missing data, researchers will try to get information by contacting the corresponding author of the referenced articles. If the author is unable to be reached, we will perform our analysis based on the available data.

#### 2.3.6 Assessment of heterogeneity

The heterogeneity of the research results will be analyzed through χ^2^ test (α = 0.1). At the same time, the heterogeneity will be determined by an *I*^2^ value. If *I*^2^ ≤50%, the statistic heterogeneity among trials can be ignored, and the effect size will be estimated using the fixed effects model. If *I*^2^ >50%, it will be considered that there is a significant heterogeneity among the trials.

#### 2.3.7 Assessment of reporting bias

When more than 10 trials are included in the study, visual asymmetry on a funnel plot will be first used to determine whether there is a publication bias. If the image is not clear, the software STATA 11.0 (College Station, Texas) will be used to perform the quantitative analysis of the Egger test.

#### 2.3.8 Data synthesis

RevMan 5.3 software (The Cochrane Collaboration, Oxford, England) will be adopted to carry out the meta-analysis. The fixed effects model will be employed for meta-analysis if there is no statistic heterogeneity among the results of the study. If there is a statistic heterogeneity, the source of the heterogeneity should be further analyzed. The random effect model will be used for meta-analysis after the effect of the obvious clinical heterogeneity is excluded. If there is obvious clinical heterogeneity, the subgroup or sensitivity analysis, or only descriptive analysis can be performed.

#### 2.3.9 Subgroup analysis

If there is a significant heterogeneity in the included trials, we will conduct subgroup analysis based on the patient's age, severity of SUI, different acupoints of the electroacupuncture, and course of treatment.

#### 2.3.10 Sensitivity analysis

On the basis of whether the adopted randomized and blind method are correct, the subgroup analysis will be performed to learn about the robustness of the meta-analysis results.

#### 2.3.11 Grading the quality of evidence

We will evaluate the quality of evidence by the Grading of Recommendations Assessment, Development and Evaluation (GRADE) and rate it into very low, low, moderate, or high 4 levels.^[19]^

## 3 Discussion

**Figure 2:**
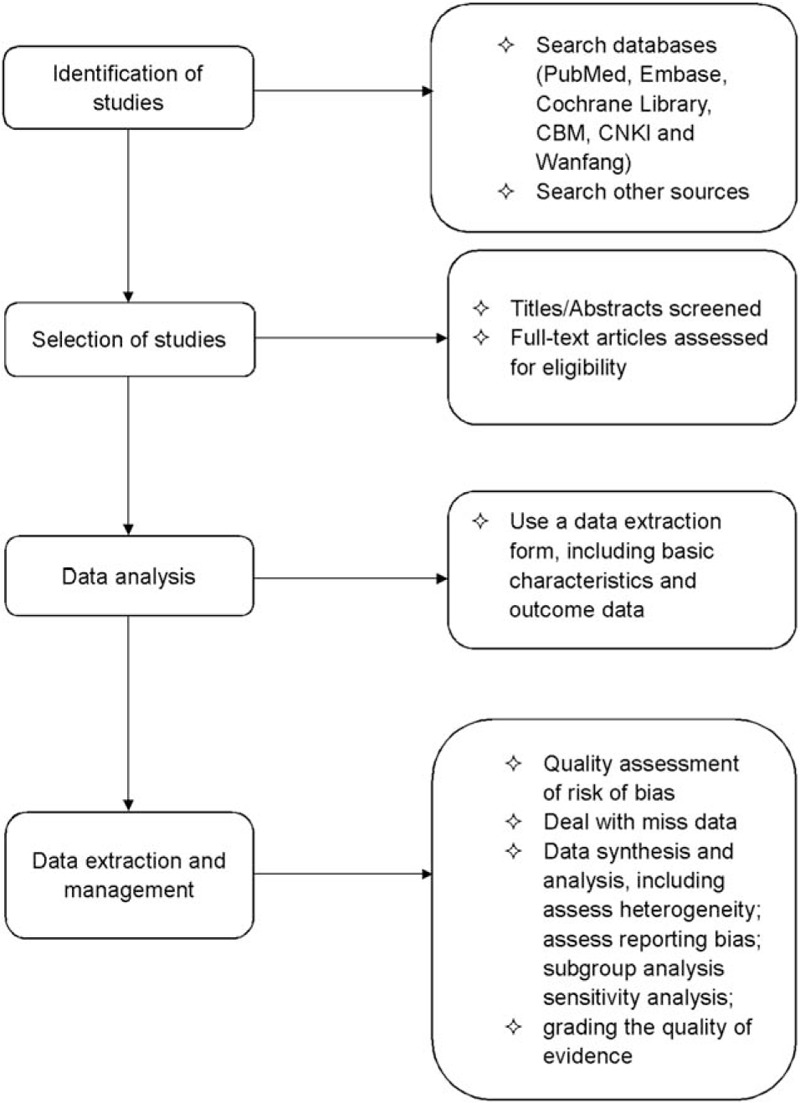
Flow diagram of the systematic review and meta-analysis.

It is essential to make sure whether electroacupuncture is a good option for the patients who are unable to have surgery, and whether it is as effective as other conservative or drug therapies. Studies have demonstrated that electroacupuncture can effectively reduce the symptoms of SUI,^[16,20]^ but its efficacy has not been evaluated scientifically and systematically. We have developed a protocol of systematic evaluation to assess the effectiveness and safety of electroacupuncture treatment on women with SUI, and the process is shown in the flow diagram in Fig. 2. The conclusions drawn from this systematic assessment will provide evidence for the policy makers, patients, and clinicians to use acupuncture for the treatment of women with SUI.

There may be some limitations in this research. Specifically, different forms of electroacupuncture and the quality of the methodologies may result in significant heterogeneity. Also, only English and Chinese medical databases will be included due to the language barrier. Hence, related studies in some other languages may be missed, such as Korean and Japanese.

## Supplementary Material

Supplemental Digital Content
