# Supplementary material for: Electroacupuncture for women with stress urinary incontinence: Protocol for a systematic review and meta-analysis
Source: Medicine (Baltimore). 2017 Dec 8;96(49):e9110. doi: 10.1097/MD.0000000000009110 (PMC5728959; doi:10.1097/MD.0000000000009110)
Supplement: Supplemental Digital Content [file medi-96-e9110-s001.docx]

**Appendix 1. Search strategy used in PubMed database**

#1Urinary Stress Incontinence OR Incontinence, Urinary Stress OR Stress Incontinence, Urinary

#2electroacupuncture OR electro-acupuncture

#3 Randomized controlled trial OR clinical study OR Clin-ical Trial OR Controlled study OR Controlled Trial OR Random*Control* study OR random* Control* Trial

#1 AND #2 AND #3
